# Diagnosis trajectories of prior multi-morbidity predict sepsis mortality

**DOI:** 10.1038/srep36624

**Published:** 2016-11-04

**Authors:** Mette K. Beck, Anders Boeck Jensen, Annelaura Bach Nielsen, Anders Perner, Pope L. Moseley, Søren Brunak

**Affiliations:** 1 Center for Biological Sequence Analysis, Department of Systems Biology, Technical University of Denmark, DK2800 Kgs. Lyngby, Denmark; 2 Novo Nordisk Foundation Center for Protein Research, University of Copenhagen, DK2200 Copenhagen, Denmark; 3 Department of Intensive Care, Rigshospitalet, University of Copenhagen, DK2100 Copenhagen, Denmark; 4 Departments of Medicine and Biomedical Informatics, College of Medicine, University of Arkansas for Medical Sciences, Little Rock, 72205 Arkansas, USA

## Abstract

Sepsis affects millions of people every year, many of whom will die. In contrast to current survival prediction models for sepsis patients that primarily are based on data from within-admission clinical measurements (e.g. vital parameters and blood values), we aim for using the full disease history to predict sepsis mortality. We benefit from data in electronic medical records covering all hospital encounters in Denmark from 1996 to 2014. This data set included 6.6 million patients of whom almost 120,000 were diagnosed with the ICD-10 code: A41 ‘Other sepsis’. Interestingly, patients following recurrent trajectories of time-ordered co-morbidities had significantly increased sepsis mortality compared to those who did not follow a trajectory. We identified trajectories which significantly altered sepsis mortality, and found three major starting points in a combined temporal sepsis network: Alcohol abuse, Diabetes and Cardio-vascular diagnoses. Many cancers also increased sepsis mortality. Using the trajectory based stratification model we explain contradictory reports in relation to diabetes that recently have appeared in the literature. Finally, we compared the predictive power using 18.5 years of disease history to scoring based on within-admission clinical measurements emphasizing the value of long term data in novel patient scores that combine the two types of data.

Sepsis is a major cause of death, contributing to almost half of deaths in hospitals^1^. Sepsis causes numerous complications, including amputations, neurological and neuromuscular disorders resulting in significant post hospital morbidity and reduced quality of life^2^,^3^,^4^. Moreover, sepsis was the most expensive condition in U.S. hospitals in 2011, costing $20.3 billion or 5.2% of the total cost for all hospitalizations^5^, emphasizing the importance of developing more cost-effective handling and care of sepsis. This includes better stratification of sepsis patients in mortality risk groups.

Simplified Acute Physiology Score (SAPS II) is a prediction model developed in 1993 and is currently used in many healthcare systems. The SAPS II score is simple and based on physiological data sampling within 24 hours of admission and three less frequent comorbidities (AIDS, hematologic malignancy and metastatic cancer). However, the medical history of patients is now increasingly becoming available in many registries and in hospital EHR systems for possible use in new and more advanced risk scores. This kind of data has for example been used to uncover temporal patterns of disease development^5^ and associations between diseases^6^. We therefore present a proof of concept for utilization of full patient disease history data collected over 18.5 years to predict 30-day mortality in patients with sepsis.

In prior work, it was demonstrated how large amounts of data from a population-wide disease registry can be condensed and organized into time dependent diagnosis trajectories that uncover recurrent, temporal disease associations^5^. The Danish National Patient Registry (NPR) coded in the International Classification of Diseases (ICD-10) terminology is one example that offers the opportunity to monitor prior disease correlations in the entire population over time. We eliminate selection biases by analyzing the full population, in contrast to for example a randomly chosen subset of patients. In our earlier use of NPR, we established time ordered disease-interrelationships, producing 1,171 recurrent disease trajectories with strong temporal directionality. Based upon these findings, we investigated whether such a time dependent, full disease history strategy could provide a useful model to predict sepsis mortality.

## Results

The overall aim was to assess the predictive value of the prehistory of sepsis patients using their full spectrum of other prior diagnoses. For this type of multi-disease analysis the patient population needs to be large in order to obtain a statistically meaningful description of the temporal diagnoses history. We therefore used a population wide registry comprising 6.6 million patients.

### Defining consistently a sepsis patient cohort

Defining the group of sepsis patients precisely is a key step, in order to eventually arrive at a meaningful mortality risk estimate. In the literature several approaches have been suggested to define groups of sepsis patients based on data in medical records coded in the ICD-9 and ICD-10 terminologies^7^,^8^,^9^. Angus *et al*. suggested one method, where patients diagnosed with infection and organ dysfunction are categorized as sepsis patients^8^. This is a widely used method, however a very recent study showed that the definition is associated with a non-optimal positive predictive value^10^. An alternative approach suggested by Ibrahim *et al*. had a significantly higher performance^7^,^10^. This approach includes most patients diagnosed with A40 (‘streptococcal sepsis’) or A41 (‘other sepsis’). In the Danish ICD-10 terminology sepsis is covered by nineteen diagnoses (Supplementary table 1), with A41 (‘other sepsis’) being the most frequently used (see supplementary text for further detail). In our earlier work on disease spectrum wide analysis of the strongest disease trajectories with the highest forward going relative risk in the Danish population, A41 appeared frequently, in fact it was included in 217 out of 1,171 recurrent trajectories computed across 6.2 million individuals^5^. We therefore initially selected the A41 diagnosis to define our sepsis population. NPR contained a group of around 120,000 sepsis patients, which had a 30-day mortality rate of 25% (eFig. 1). When counting our patients using the ‘Ibrahim implementation’, 119,727 sepsis patients were shared between our method and the ‘Ibrahim implementation’, our definition covered an additional 112 patients, whereas the ‘Ibrahim implementation’ covered an additional 6,839, and this simple approach is therefore very similar to the ‘Ibrahim implementation’. Consequently, it will have very similar properties in sensitivity analyses. In this type of work it is most important to ensure that all patients classified as sepsis patients actually have sepsis, while it will not affect the results in any major way if we potentially miss a small number of patients who also had sepsis (as the control population without sepsis amounts to more than 6 million individuals).

### Sepsis mortality altered by 231 diagnoses

Prior to analyzing risk associated with multiple diagnoses, we investigated to what extent single diagnoses in the sepsis patients’ prior disease history significantly changed the 30-day mortality. Of 1,051 level three ICD-10 diagnoses (with a minimum number of patients counts required for statistical analysis, see Methods) 231 diagnoses significantly changed the relative risk of dying from sepsis (RR_sepsis dead_). See eFig. 2 and supplementary text for further detail.

### Different temporal disease trajectories observed within the sepsis population

Analyzing correlations between many earlier diagnoses may obviously give a better indication of a patient’s health status. We investigated how sequences of consecutive diagnoses (disease trajectories) changed RR_sepsis dead_ to obtain a more comprehensive understanding of the association between prior disease history and sepsis mortality risk. Temporal disease trajectories were constructed by finding all significantly associated diagnoses in pairs and by selecting those with a temporal direction (where one disease occurred significantly more often before the other). These temporal disease pairs were then combined into trajectories of temporal consecutive diseases. In the case of four disease steps, when disease A was preceding disease B, B was preceding C, and C was preceding D we would have three temporal disease pairs and a disease trajectory containing diseases A, B, C and D in that order (see Methods).

In our sepsis population of approximately 120,000 patients, we found 2,279 such disease trajectories consisting of four consecutive diseases, where a minimum of 20 patients followed the entire trajectory. In all these trajectories sepsis appeared exclusively as the fourth and last diagnosis, implying that there are many disease paths towards sepsis. However, within the cutoff values applied we did not find any trajectories continuing after sepsis, indicating a much less systematic pattern of comorbidities occurring after sepsis.

Out of 120,000 sepsis patients in our cohort, 28,484 followed at least one of the 2,279 time-dependent disease trajectories. As there might be some discrepancy between time of clinical representation and date of diagnosis, following the trajectories in a strict chronological order might exclude patients with very similar disease courses. Relaxing the criterion such that certain pairs of diagnoses could appear in different orders (as long as sepsis was the last diagnosis) 40,247 sepsis patients followed at least one of these trajectories.

### A subset of 56 temporal disease trajectories predicted poorer health status

**Figure 1:**
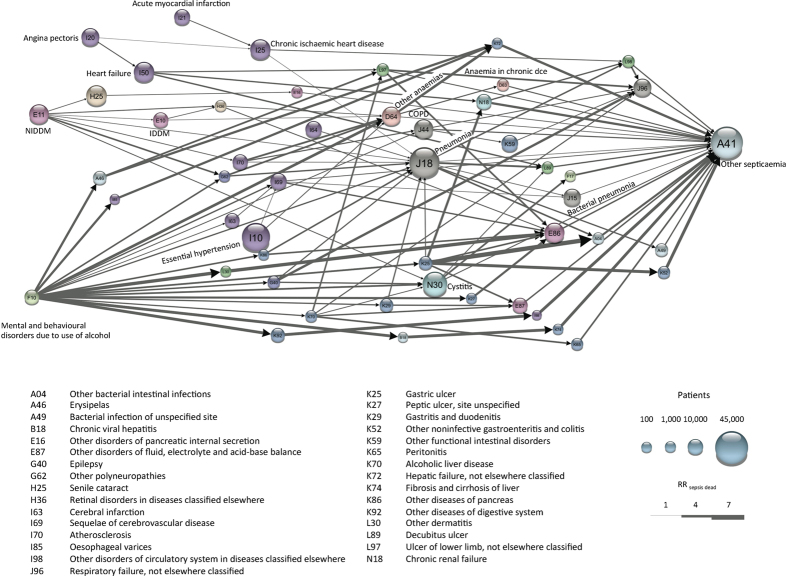
Network of trajectories that significantly altered RR_sepsis dead_. The network was constructed from 56 significant sepsis trajectories and illustrates simultaneously the number of patients receiving a particular diagnosis (node size) and the increased risk of dying from sepsis within 30 days from different trajectory steps connecting two diagnoses (width of arrow). The 42 nodes are colored based on their ICD-10 chapter relationships. Note that A41 has been scaled to 33% of its actual size representing 120,000 patients. The width of the arrows indicates the weighted average RR_sepsis dead_ for a particular step (based on all trajectories containing that step).

We calculated RR_sepsis dead_ for patients following at least one trajectory compared to sepsis patients not following any trajectory to investigate the predictive value of the trajectories for mortality in these patients. The RR_sepsis dead_ was calculated using the Cochran-Mantel-Haenzel method, which allows us to correct for age and gender. The RR_sepsis dead_ for the 28,484 patients following at least one trajectory strictly in the right order was 1.32, (p = 1.88·10^−77^). RR_sepsis dead_ was 1.30 (p = 2.46·10^−82^) for the 40,247 patients following trajectory diagnoses in the relaxed order, indicating the ability of the disease trajectories to be predictive of a poorer health status in a sepsis patient. The age of patients following a trajectory was significantly higher both for the strict and relaxed orders (odds ratio = 2.3 and 2.4 respectively, p-value < 2.3·10^−308^ for both, correction for age in the mortality analyses). We subsequently made a network based on all trajectories that significantly changed the risk of dying (Benjamini Hochberg corrected p-value < 0.05) reducing the number of trajectories from 2,279 to 56 (Fig. 1). The network is constructed from 97 unique disease pairs (and edges connecting them) that appear in any of the steps in the 56 trajectories. All individual trajectories increased RR_sepsis dead_ clearly underlining the ability of the trajectories to predict a poorer outcome. Of the 56 significant trajectories 25 started with alcohol abuse, nine contained diabetes mellitus, while eighteen contained cardiovascular diagnoses.

### Alcohol abuse, diabetes and anemia are key diagnoses in the sepsis trajectory network

**Figure 2:**
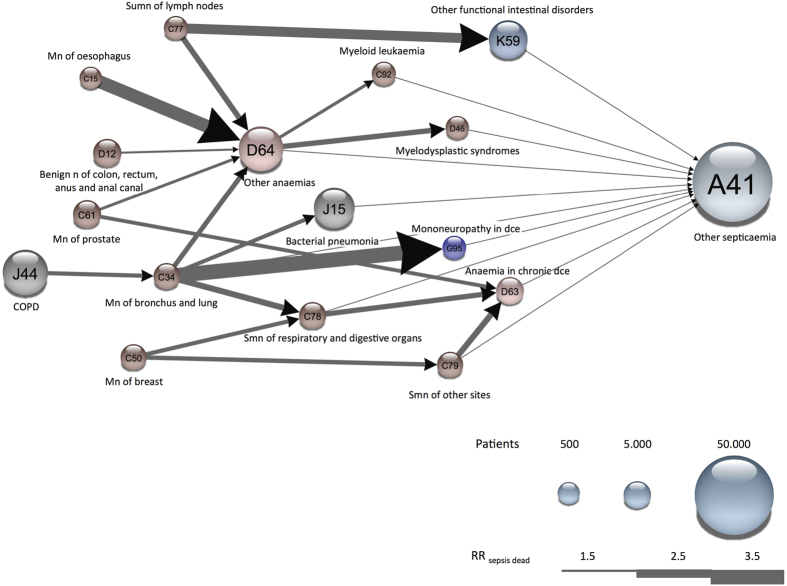
Cancer-sepsis network from length three trajectories that significantly altered RR_sepsis dead_. The network was created from sixteen length three sepsis-trajectories, which all contain a minimum of one disease from the ICD-10 block “Cancers (C00-C96)” from ICD-10 chapter 2: Neoplasms. The nodes are colored based on their ICD-10 chapter. Their size corresponds to the number of sepsis patients having the particular diagnosis. The width of the arrows indicates the RR_sepsis dead_ for a particular step in a trajectory.

Figure 1 shows all 56 significant trajectories towards sepsis in one network. It has the three major starting points mentioned above: ‘alcohol abuse’, ‘non-insulin dependent diabetes mellitus’ (NIDDM) and cardio-vascular diagnoses (‘angina pectoris’, ‘acute myocardial infarction’ and ‘hypertension’). A number of other diagnoses appear frequently, including ‘other anemias’. No cancer diagnosis appeared in the network although the RR_sepsis dead_ for patients having any neoplasms (benign or malignant) prior to the sepsis diagnosis was 1.24 (p = 1.2⋅10^−60^) when calculated across the 120,000 patients and 1.18 (p = 4.97⋅10^−30^) for patients having any cancer before sepsis (eTable 1). Patients can be healthy until diagnosed with cancer^11^, which then may trigger sepsis. Their shared disease history might therefore be less similar compared to diabetes patients, which can explain the lack of fine-grained cancer trajectories of four diseases. We found, however, seventeen cancer trajectories out of 310 trajectories consisting of only three diseases with sepsis as the last diagnosis. A cancer-network of these trajectories also revealed that anemia was the major connection between cancer and sepsis (Fig. 2). This further indicates that the absence of length four cancer trajectories was due to a lack of shared disease history rather than insignificant effect on sepsis mortality.

**Figure 3:**
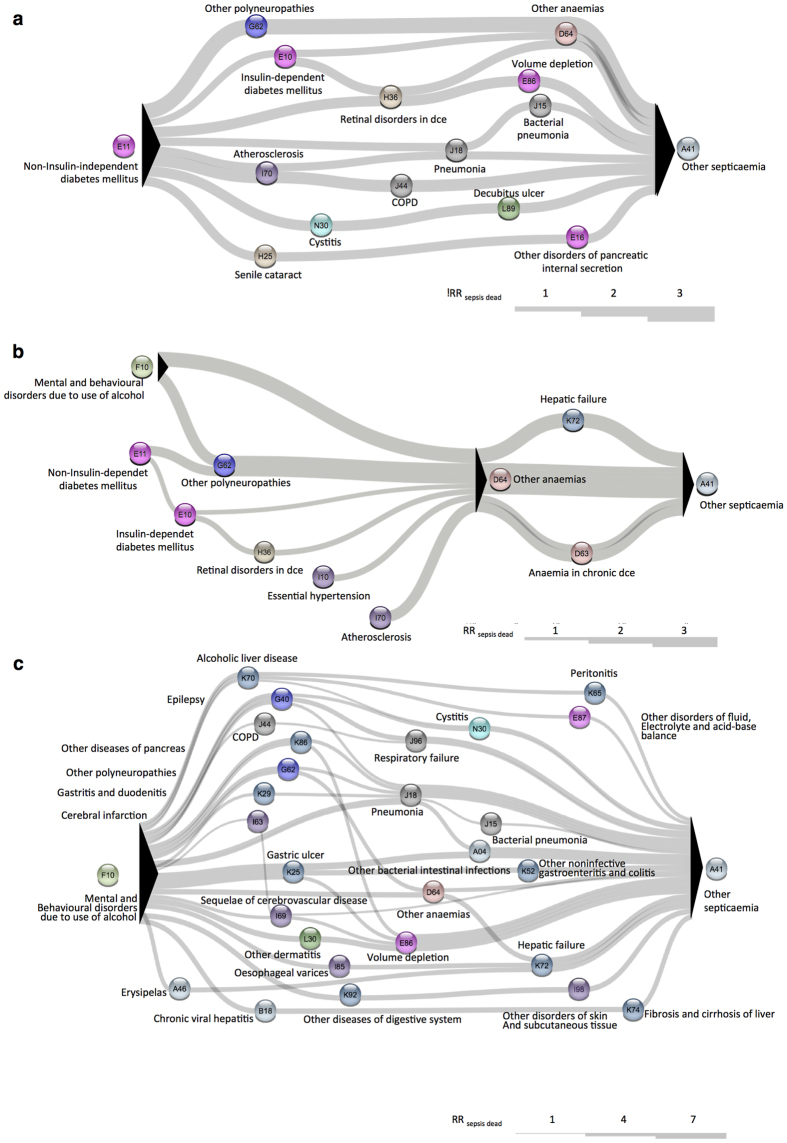
Sepsis sub-networks from trajectories that significantly altered RR_sepsis dead_. The three networks were constructed from the 56 significant sepsis trajectories described in Fig. 1. A continuous line illustrates a group of patients following a specific multi-step trajectory. This includes all trajectories that contains either (**a**) Insulin-dependent or insulin-independent diabetes mellitus, or (**b**) Other Anaemias or Anaemia in chronic diseases classified elsewhere, or (**c**) Mental and behavioural disorders due to use of alcohol. The nodes are colored according to their ICD-10 chapter. The width of the arrows indicates the RR_sepsis dead_ for a particular trajectory.

We investigated ‘alcohol abuse’, ‘diabetes mellitus’ and anemia in more detail by extracting trajectories containing these particular diagnoses (Fig. 3). Due to the limited number of trajectories included in the Fig. 3 illustrations, we have shown the exact trajectory for a complete patient group, by having one (curved) edge connecting all four diseases. We observed a handful of alternative routes from diabetes to sepsis, via decubitus ulcers, pneumonia, anemia, and volume depletion (Fig. 3a). All trajectories in the diabetes network started with NIDDM followed by two additional comorbidities before sepsis. The highest risk was associated with ‘other polyneuropathies’ followed by ‘pneumonia’, but also atherosclerosis and subsequently COPD implied high sepsis-mortality risk (Fig. 3a).

The temporal disease trajectories in the network starting with ‘alcohol abuse’ were much more diverse than those in the diabetes network. Many of these pathways included common complications of alcoholism like ‘diseases of the digestive system’, ‘epilepsy’, ‘cerebral infarction’ and polyneuropathies (Fig. 3b).

Anemia is seen frequently in pre-menopausal women, however the age and gender distribution rule this out as the main cause in our population (eFig. 3). For all trajectories in the anemia network, anemia was the last diagnosis before sepsis. The anemia-sepsis network revealed the same pattern of starting points as the complete sepsis network, including vascular diseases, diabetes and alcohol abuse. Trajectories including vascular diseases also had the two anemia diagnoses before sepsis. The diabetes trajectories had diabetic comorbidities before anemia and subsequently sepsis. Lastly, one of the trajectories starting with ‘alcohol abuse’ had anemia before ‘hepatic failure’.

### Comorbidities explained the differences in sepsis mortality reported in diabetes

RR_sepsis dead_ for patients with diabetes has been debated recently in the literature with studies indicating both higher and lower mortality risk^12^,^13^,^14^,^15^. In our study ‘insulin dependent diabetes mellitus’ (IDDM) increased the risk (RR_sepsis dead_ = 1.13, p = 1.77·10^−30^), whereas NIDDM had no effect (RR_sepsis dead_ = 1.11, p = 0.15). When combining IDDM and NIDDM, the RR_sepsis dead_ was 1.11 (p = 5.71·10^−9^) underlining the important difference in strength of association of IDDM and NIDDM in regard to survival of sepsis.

**Figure 4:**
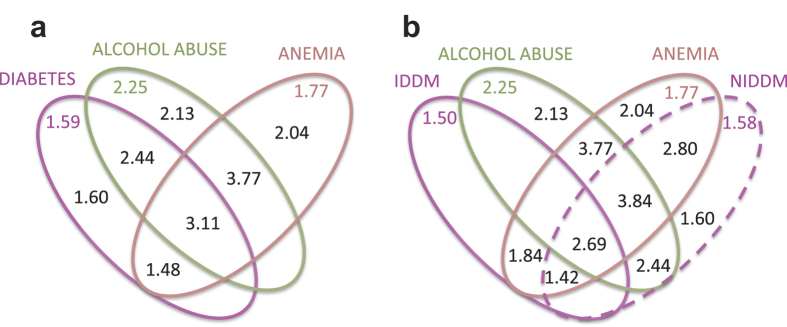
Venn diagrams of selected sepsis-trajectories that significantly altered RR_sepsis dead_. The Venn diagrams show the significant RR_sepsis dead_ values for groups of patients following trajectories containing diabetes mellitus (E10, E11), alcohol abuse (F10) and/or anemia (D64), respectively. The colored digits (inside and outside the three ellipsoids) indicate RR_sepsis_ dead for following a trajectory that contains that particular diagnosis, independently of which of the other trajectories the patient follows. Ellipsoids without values are insignificant.

Our trajectories included nine diabetes trajectories that significantly altered RR_sepsis dead_, eight NIDDM trajectories, one IDDM diabetes trajectory and one containing both IDDM and NIDDM. All nine trajectories increased the risk of death with an RR_sepsis dead_ between 1.45 and 3.01. Besides diabetes, ‘alcohol abuse’ and ‘other anemias’ were two other main diagnoses in our network (Fig. 1). We calculated the RR_sepsis dead_ associated with any disease trajectories containing certain specific diagnoses (Fig. 4a). Following a diabetes mellitus trajectory was associated with an RR_sepsis dead_ of 1.59 (p-value = 6.27·10^−23^), but combined with an alcohol abuse trajectory or an anemia trajectory RR_sepsis dead_ increase to 2.44 (p = 5.83·10^−4^) and 1.48 (p = 1.98·10^−4^), respectively. If the patient followed all three types of trajectories RR_sepsis dead_ was 3.11 (p = 4.44·10^−6^). Separating diabetes in IDDM and NIDDM showed a higher degree of synergy between anemia and NIDDM (RR_sepsis dead_ = 2.80) than anemia and IDDM (RR_sepsis dead_ = 1.84) (Fig. 4b).

### Independent replication using a Swedish comorbidity browser

**Figure 5:**
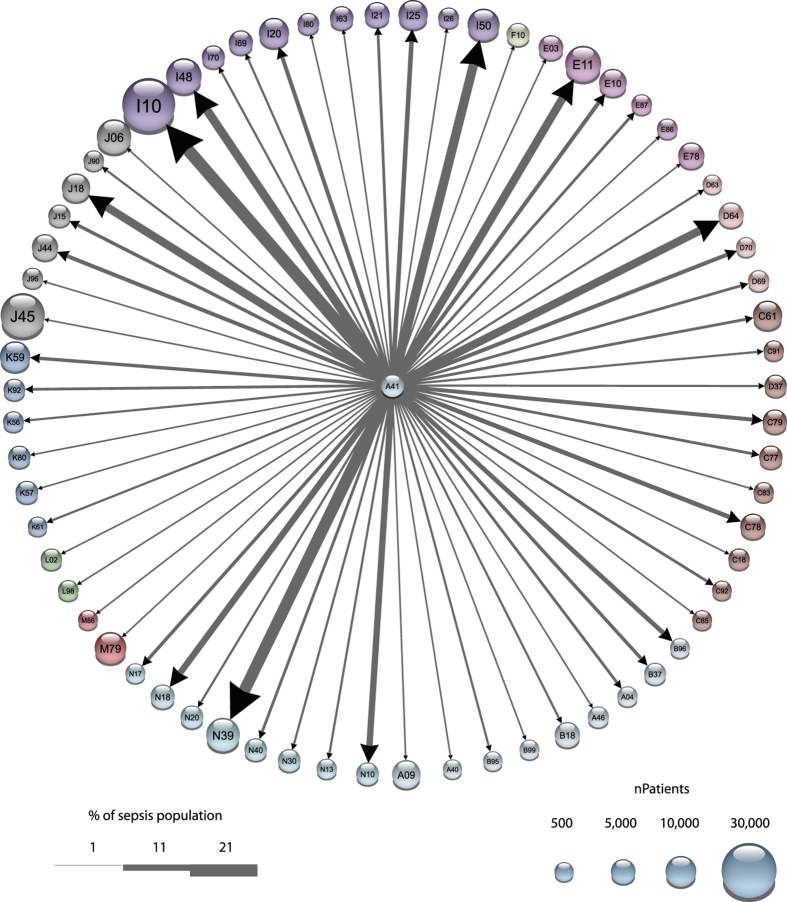
Sepsis comorbidities in the Swedish study. This figure shows significant co-morbidities in the Swedish study, Dalianis *et al*.^16^. Node size corresponds to the number of patients in the Swedish cohort with that code. The width of the arrow indicates the percentage of the sepsis subpopulation with a particular comorbidity. The lengths of the radiating arrows are arbitrary.

We compared the sepsis comorbidities found in our data, to a time-independent comorbidity browser made by Dalianis *et al*.^16^. The browser is based on 600,000 Swedish patients and use also the ICD-10 terminology^16^. Although the browser did only provide access to summary level data (and no individual level data) we replicated all three major starting points in the main network of significant trajectories, both types of diabetes mellitus, all four cardiac diseases (I20, I21, I25 and I50) as well as ‘alcohol abuse’ were sepsis comorbidities in the Swedish study (Fig. 5). We further confirmed the two anemia codes (D63 and D64) observed in the network. Additionally, we confirmed that several cancers were significantly associated comorbidities, although the number of patients for each individual cancer within the sepsis population also was relatively small in the Swedish data set, which again can explain the lack of length four cancer-disease trajectories in our data. Overall, our main observations of comorbidities were confirmed in this independent cohort.

## Discussion

Contrary to conventional epidemiological techniques focusing on a few diagnoses, we used a multi-morbidity starting point, allowing us to identify more than 2,200 temporal disease trajectories of four consecutive diseases found in sepsis patients. We reduced the 2,220 trajectories to 56 that significantly change the risk of dying from sepsis within 30 days after diagnosis. We used data covering 120,000 sepsis patients followed over 18.5 years of disease history making it significantly larger both in terms of the number of patients and time than other earlier, classical mortality studies in sepsis or ICU patients (e.g. MPM-II, SAPS II and APACHE-II with 19,124; 13,000; and 805 patients, respectively)^17^,^18^,^19^. To our knowledge this is the first study of this size that uses all diseases from a long time period to determine the specific mortality risk for sepsis patients. Our data demonstrates the important predictive value of this disease trajectory approach accounting for time-ordered combinations of prior diseases to determine mortality risk.

It is important to notice that the temporal disease trajectories consist of disease associations with significant temporal directionality only. However, this cannot be used to conclude that one disease causes the second disease, as many confounding factors can influence the associations.

Although this type of analysis is based on disease spectrum-wide principles, the results are easily incorporated into mortality risk scores used in the clinic, as the patient data is already stored electronically and accessible in well-organized registries.

We demonstrated that single diseases or minor groups of diseases (as used in SAPS II) comparatively only hold limited predictive value about the patient’s health status. Here we investigated the effect of essentially all diseases coded in the ICD-10 system in a data driven manner. Several secondary cancers scoring an RR_sepsis dead_ between 1.5 and 2 corresponded well with the fact that metastatic cancer is one of the three chronic diseases among the seventeen factors included in the SAPS II, which predicts hospital mortality^17^. The SAPS II converted value of metastatic cancer corresponds to an RR of 1.08 (see Methods), a substantially lower RR than observed in our population-wide data.

The second chronic disease in SAPS II, ‘HIV positive with an AIDS defining illness’, has an RR of 1.15. In our data set this was covered by the four diseases within the block ‘Human immunodeficiency virus [HIV] disease’ none of which significantly change the RR_sepsis dead_. This may be due to a relatively small group of patients as well as the considerable improvement in HIV treatment in recent years. The third and last chronic disease in the SAPS II score is hematologic malignancies with a calculated RR of 1.09 (ICD-10 codes C81-C96), corresponding to the block ‘Malignant neoplasms, stated or presumed to be primary, of lymphoid, haematopoietic and related tissue’. Of these fourteen diagnoses four significantly changed the RR_sepsis dead_ in our dataset. However, RR_sepsis dead_ varied from 1.5 to 2.3 (Supplementary Table 2). It should also be noted that SAPS II calculates the mortality of all ICU patients, whereas we investigated sepsis patients only.

A logical follow on to the work presented here is to combine RR_sepsis dead_ with physiological parameters and make an updated mortality score. The original idea behind SAPS II was to avoid non-physiological values such as previous diseases, largely because the diagnostic history was not available electronically. However, using computationally ready datasets such as the Danish health registry, many of these prognostic scoring systems may be improved by taking into account temporal aspects of disease history. We show that this undeniably has an effect on a patient’s outcome. For all countries with an electronic disease registration scheme and personal identification numbers that can track patients over time, this could in a feasible manner be transferred for use in a revised clinical score.

In addition to the potential direct impact of prior disease history on individual patient outcomes in sepsis, our findings imply that studies of sepsis, and specifically interventional studies, may fail to show differences in treatments because of a lack of appropriate stratification schemes. Recently, two large, multicenter, randomized trials of protocol driven care (ProCESS^20^ and ARISE^21^) failed to demonstrate the superiority of protocol based care over usual care. Both studies controlled for comorbidity effects largely using a well-established epidemiology based comorbidity index built on outcomes of 226 individuals^22^,^23^. Many arguments have been proposed for the lack of difference in outcomes between the two groups. Our data offer a potentially important opportunity to consider the role of complex full disease history based upon large population analysis in patient group assignment.

Specifically, it has been discussed whether diabetes increases or decreases the risk of dying from sepsis^12^,^13^,^14^,^15^. In most papers, the authors do not distinguish between IDDM and NIDDM, which was possible for us due to the size of the data set. Our analysis shows clearly that there is a distinct difference in sepsis mortality for IDDM compared to NIDDM if patients also follow a disease trajectory containing anemia (RR_sepsis dead_ of 1.84 and 2.80, respectively). This implies a higher degree of synergy between NIDDM and anemia than between anemia and IDDM. Our findings suggest therefore that both types of diabetes and temporal combinations of comorbidities must be corrected for when determining sepsis mortality in diabetes patients. This may explain the reported discrepancy of sepsis mortality in diabetes patients.

Even though we investigated these relationships in a temporal manner, it is again important to notice that temporal association does not necessarily imply causation. There can be many confounding factors explaining these associations such as occupation, socioeconomic status or life style choices, which the diagnoses data does not cover.

Collectively, we show the importance of the previous disease history for predicting the outcome of sepsis patients. We suggest that the temporal disease history should be an additional aspect of personalized medicine, as we showed its significant value here. We further suggest that temporal disease history should be implemented as a stratification parameter in clinical trials, as we in this study demonstrated its application for prediction the sepsis mortality in diabetes patients.

## Materials and Methods

### Study design

In this retrospective cohort study we use a data-driven approach to identify mortality risk groups within a sepsis population based on a population-wide disease registry, which contains administrative information and primary and secondary diagnoses coded using ICD-10 terminology and used for reimbursement purposes. We used a dataset from the Danish National Patient Registry, which covers all hospital encounters (inpatient admissions, outpatient visits and emergency room visits) in Denmark from 1st of January 1996 until 19th of August 2014, collectively counting 6.6 million patients of which almost 120,000 were diagnosed with A41 ‘Other sepsis’, of which almost 30,000 acquired sepsis after admission. The ICD-10 system is structured hierarchically, where codes can be rounded to a less specific parent diagnosis code, block or chapter. We used this structure to round all codes to level three codes. Our aim was to identify temporal disease trajectories derived using pairs of significant time-dependent diagnosis correlations, which significant change the 30-day mortality risk, based on the last sepsis diagnosis for each patient.

### Mortality risk assessment

We tested if a single disease or trajectory from a patient’s prior disease history altered the mortality risk in sepsis patients. The relative ratio of dying from sepsis was calculated using the Cochran–Mantel–Haenszel method (an advanced version of Fischer’s exact test, where data can be binned to correct for co-variants), where each bin correspond to patients of a particular gender and born in a particular decade. We included patients born from 1900 until 2014, giving rise to up to 24 bins per test. We used the Cochran–Mantel–Haenszel test to identify the p-value and accepted results with a Benjamini-Hochberg corrected p-value of 0.05 or below.

### Temporal diagnosis pairs, sepsis-trajectories and network

The method for identifying the trajectories was described previously in detail^5^. The method consists of three steps: First 826,427 temporal directed pairs of co-morbid diseases were tested to identify pairs where one disease increases the risk of later occurrences the other. In the second step, a set of 4,005 diagnoses that had a significant order (one disease primarily occurring before the other) was identified. It should be noted that the comorbidities were recalculated on a refined version of the original dataset where some minor issues with discharge dates were fixed. Third, this set of pairs was combined into longer trajectories of temporal consecutive diseases. In the case of four disease steps, when disease A was preceding disease B, B was preceding C, and C was preceding D we would have three temporal disease pairs and a disease trajectory containing diseases A, B, C and D in that order. We required a minimum of 20 patients to follow the entire trajectory. We combined temporal trajectories into disease networks graphs, by importing all disease pairs included in the trajectories into a network visualized by Cytoscape. The x-axis in the networks represent “time-order” and implicitly time although the x-axis is not time-true as progression from one disease to another vary across individuals and across disease pairs. It is therefore not possible to draw a compact time-true plot in two dimensions.

### Calculating age differences

We calculated the difference in age using a general linear model, including gender and year of birth as covariates.

### Validation of co-morbidities

We compared the sepsis co-morbidities (any disease defined as level three diagnoses in the ICD-10 terminology occurring in patients with sepsis) found in the Danish NPR with the co-morbidities of A41 in a Swedish co-morbidity browser, based on almost 600,000 individuals from the greater Stockholm area: http://www2.dsv.su.se/comorbidityview-demo/.

### Calculating RR in SAPS II

To calculate the probability of in-hospital mortality according to SAPS II, we used an equation developed and published earlier (Equation 1)^17^ and converted it into a probability (Equation 2):


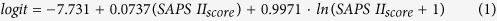



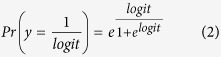


The SAPS II_score_ ranges from 0pt to 163pt. Thus, by employing the equations above, the logit of every integer between 0 and 163 was converted to probabilities and summed to obtain the area under the curve (AUC) for the “baseline” SAPS II_score_. In a similar manner probabilities for the three comorbidities metastatic cancer, hematologic malignancy and AIDS were computed with the only difference being the addition of each their weights to the baseline SAPS II_score_; 9 points, 10 points and 17 points, respectively. The relative risks were then found by dividing the comorbidity AUC’s to the baseline SAPS II AUC.

### Data and materials approval

This study was approved by Danish Data Protection Agency, Copenhagen (ref: 2010–54–1059) and Statens Serum Institut (ref: FSEID-00001136).

## Additional Information

**How to cite this article**: Beck, M. K. *et al*. Diagnosis trajectories of prior multi-morbidity predict sepsis mortality. *Sci. Rep.* **6**, 36624; doi: 10.1038/srep36624 (2016).

**Publisher’s note:** Springer Nature remains neutral with regard to jurisdictional claims in published maps and institutional affiliations.

## Supplementary Material

Supplementary Information
