# Supplementary material for: Diagnosis trajectories of prior multi-morbidity predict sepsis mortality
Source: Sci Rep. 2016 Nov 4;6:36624. doi: 10.1038/srep36624 (PMC5095673; doi:10.1038/srep36624)
Supplement: Supplementary Information [file srep36624-s1.pdf]

Diagnosis trajectories of prior multi-morbidity predict sepsis mortality

Mette K. Beck, Anders Boeck Jensen, Annelaura Bach Nielsen, Anders Perner, Pope L. Moseley, Søren Brunak

Supplementary text

Results

231 diagnoses altered the sepsis mortality

As expected we observed differences both among and within ICD-10 chapters. Diagnoses associated increasing $RR_{sepsis\ dead}$ the most were in particular observed within the chapters: 'Certain conditions originating in the perinatal period', 'Congenital malformations, deformations and chromosomal abnormalities', 'Endocrine, nutritional and metabolic diseases' and 'Neoplasms'. In the other end of the spectrum, most diagnoses within 'Diseases of the musculoskeletal system and connective' and 'Diseases of the genitourinary system' were associated with decreased $RR_{sepsis\ dead}$. Among all chapters, the unspecific 'Factors influencing health status and contact with health services' displayed a large variation in $RR_{sepsis\ dead}$. The top seven diseases shown in eFigure 2 are within 'Diseases in the perinatal periods'. They have an $RR_{sepsis\ dead}$ spanning from 9.7 to 27.7 and are followed by four diseases from 'Congenital malformations, deformations and chromosomal abnormalities'. These likely represent miscoding, and should have been assigned P36 'Bacterial sepsis of newborn' rather than A41. Subsequently, the hormonal and metabolism related diagnoses 'Disorders of glycoprotein metabolism' and 'Other ill-defined and unspecified causes of mortality' top the list with an $RR_{sepsis\ dead}$ of 5.2 and 5.9 (p-values = $4.69 \cdot 10^{-2}$ and $1.65 \cdot 10^{-31}$), respectively. Interestingly, all malignant cancer diagnoses score an $RR_{sepsis\ dead}$ between 1.5 and 2.8, including several secondary malignant neoplasms. 'Anemias in diseases classified elsewhere' and 'Other Anemias' had an $RR_{sepsis\ dead}$ of 1.6 and 1.5 (p-values = $2.96 \cdot 10^{-35}$ and $2.51 \cdot 10^{-110}$), respectively. Insulin-dependent diabetes mellitus (IDDM) had an $RR_{sepsis\ dead}$ of 1.13

(p-value = $2.72 \cdot 10^{-4}$), whereas the value for non-insulin-dependent diabetes mellitus (NIDDM) was 1.11 (p-value = $5.33 \cdot 10^{-6}$).

Sepsis patients stratified into more than 2,200 different trajectories

We observed a long tailed distribution, where a few trajectories were followed by many patients, while most trajectories were followed by fewer patients. We found that 21 of the top 45 trajectories belonged mainly to 'Diseases of the circulatory system', confirming that many sepsis patients had chronic cardio-vascular problems (eTable 2). 3,366 patients followed the most common trajectory, where patients had 'Angina Pectoris' before 'Chronic ischemic heart disease' with 'Pneumonia' third. Another 15 of the top 45 trajectories belonged to the 'Endocrine, nutritional and metabolic diseases' chapter. More specifically, these 15 trajectories all contained two or three of the diabetes ICD-10 codes ('IDDM', 'NIDDM', and 'unspecific diabetes mellitus'), confirming, that many patients with sepsis had diabetes mellitus prior to their sepsis diagnosis.

Many patients followed several pathways, especially trajectories containing similar diagnoses within the same chapter. We therefore decided to examine the number of patients that followed each trajectory in a manner where each patient only was counted in the most populated trajectory he/she followed thereby visualizing better the diversity across the sepsis population.

We then observed several new chapters occurring, including 'Diseases of the genitourinary system', 'Diseases of the respiratory system', 'Neoplasms', 'Diseases of the blood and blood-forming organs and certain disorders involving the immune

mechanism' and 'Diseases of the musculoskeletal system and connective tissue'. This again indicated that patients getting sepsis were relatively diverse segregating into several major, but largely independent patient sub-groups.

Discussion

Mortality measure

30-day mortality is the most widely used measure, although some studies use in-hospital or 90-day mortality. The in-hospital and 30-day mortality for sepsis patients are similar (*24*), whereas the 90-day mortality may include late adverse effects of sepsis and related therapies (*25*). For simplicity, we chose 30-day mortality for this analysis.

Unspecific diagnosis

In our data, unspecific diagnoses like "Other anemias", "Other septicaemia" and "Bacterial pneumonia, not elsewhere classified" were much more frequently used than specific diagnoses like "Anemia in chronic diseases classified elsewhere", "Streptococcal sepsis" or "Pneumonia due to Streptococcus pneumonia". This might be explained by the fact that specific diagnoses often require more and longer patient examination, but it may also be that doctors have to assign diagnoses outside of their main area of expertise.

Analyzing registry data containing 120,000 sepsis patients can lead to extremely low p-values, which proves a significant difference in the mean of the groups. However, it is important to emphasize the importance of the change in sepsis mortality rather than only focusing on the significance level. Sepsis patients with 'anemias in diseases classified elsewhere' or 'other Anemias' had an $RR_{sepsis\ dead}$ of 1.6 and 1.5 (p-values =

$2.96 \cdot 10^{-35}$ and $2.51 \cdot 10^{-110}$), respectively. The difference in p-value is uninteresting, whereas the similar change in mortality indicates an equal medical relevance.

We confirmed the pattern of comorbidities found in the Danish data by comparing it to a Swedish comorbidity study, using similar Swedish electronic patient records[8]. The Swedish cohort was significantly smaller (3,409 sepsis patients and 600,000 patients overall), but confirmed the comorbidities found in our main trajectory network (eFigure 4).

Figures

eFigure 1. Survival curve for sepsis patients. The survival curve shows the survival ratio for patients diagnosed with A41.

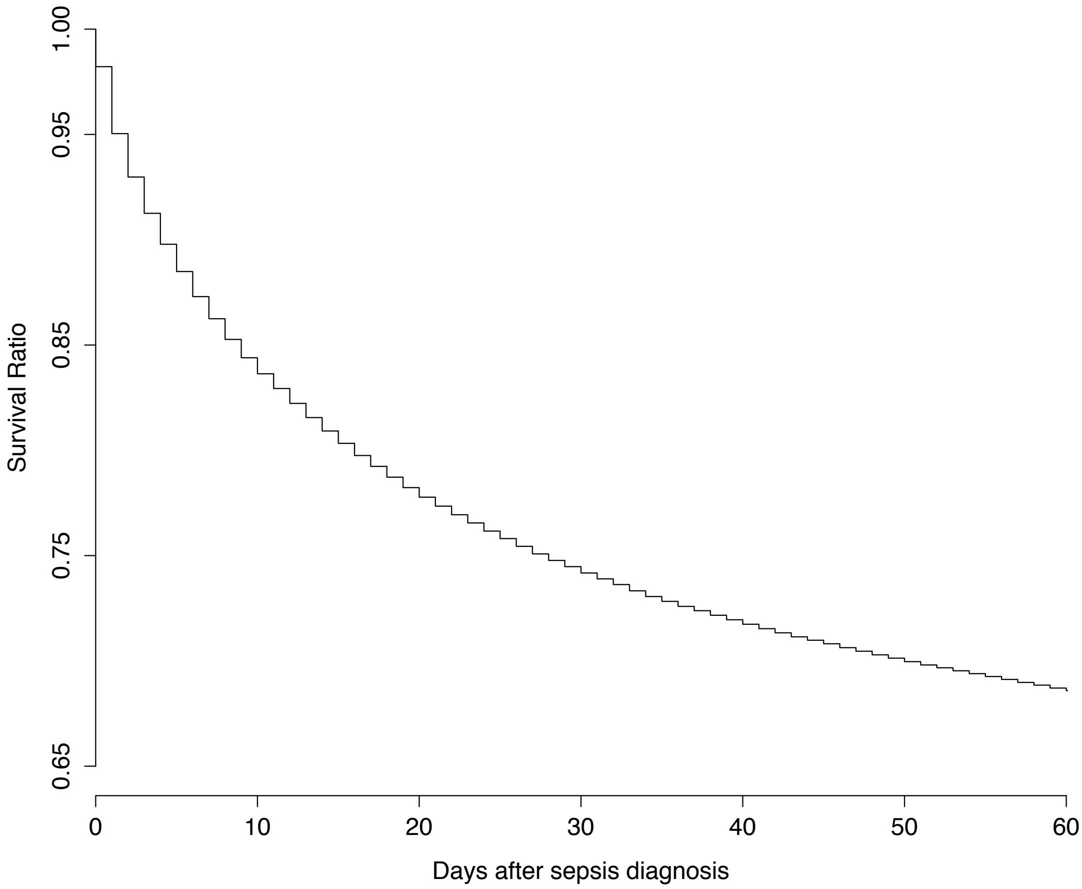

eFigure 2. Prior individual diagnoses significantly changed the risk ratio of dying from sepsis within 30 days. Each dot represents an ICD-10 level 3 diagnosis acquired before the sepsis diagnosis (A41).

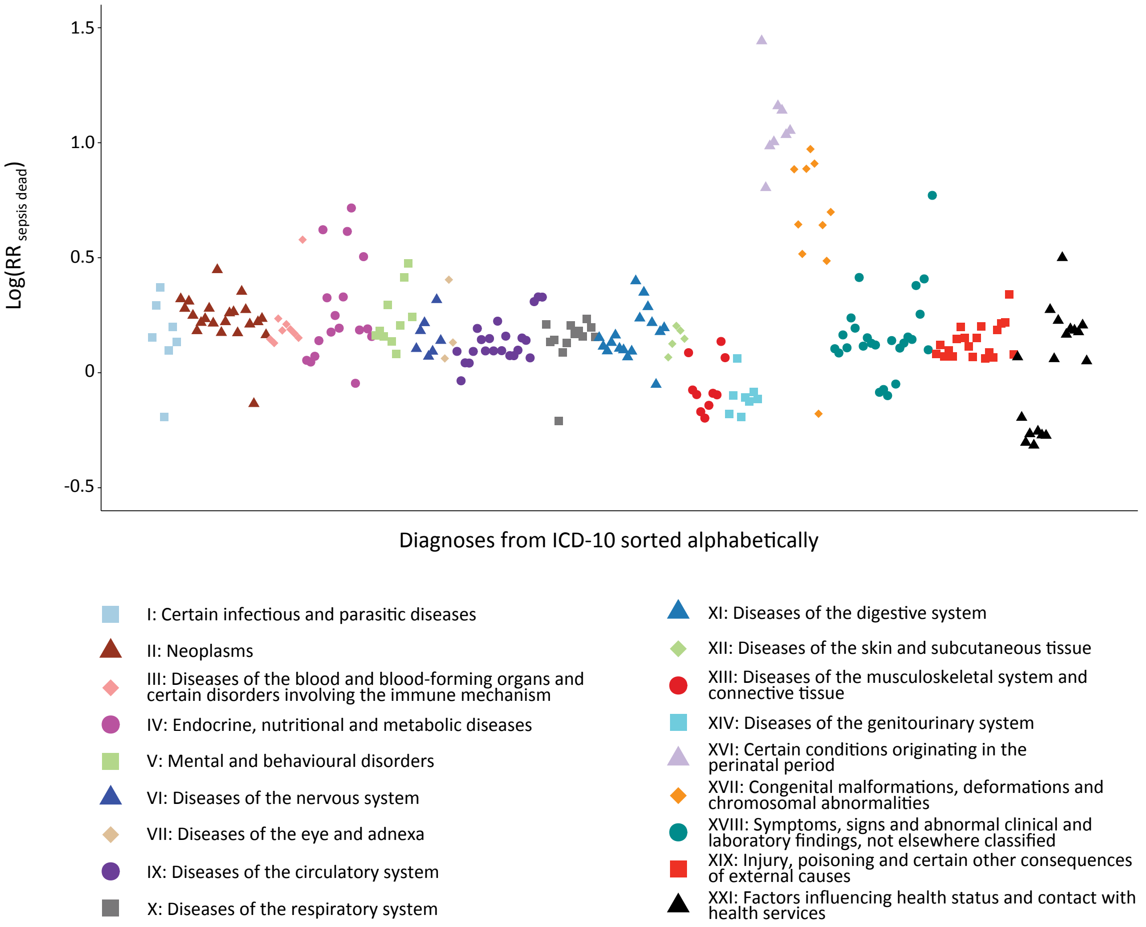

eFigure 3. Age and gender distribution for the most common anaemia diagnosis (D64). The plot shows the age distribution for the onset of anemia in male (blue) and female (red) patients. Both groups peak in the early eighties.

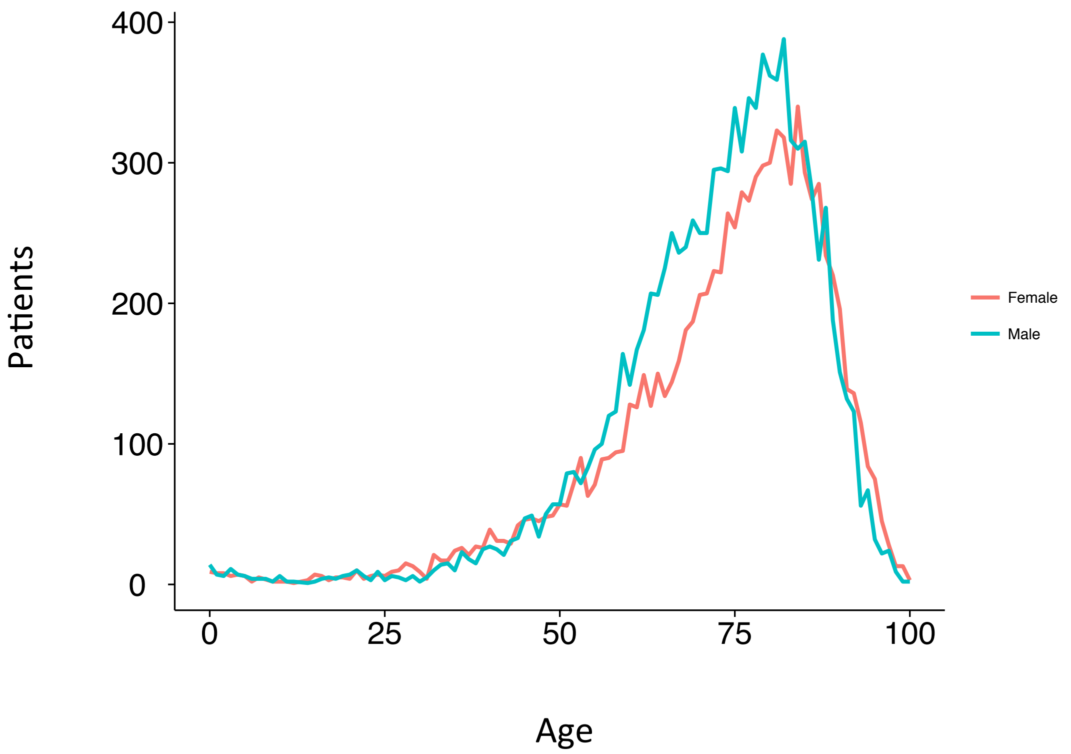

eTable 1. List of neoplasms, stated or presumed to be primary, of lymphoid, haematopoietic and related tissues. The table indicates significant $RR_{sepsis\ dead}$ values for patients having had one of these cancer diagnoses previously and the corresponding Benjamini-Hochberg corrected p-value.

ICD - 10	Disease	$RR_{sepsis\ dead}$	BH corrected P-value	Patient count
**C81**	Hodgkin lymphoma	0.87	0.73	814
**C82**	Follicular lymphoma	0.85	0.31	887
**C83**	Non-follicular lymphoma	0.73	$2.31 \cdot 10^{-9}$	2,551
**C84**	Mature T/NK-cell lymphomas	1.10	0.93	476
**C85**	Other and unspecified types of non-Hodgkin lymphoma	0.86	0.01	2,659
**C86**	Other specified types of T/NK-cell lymphoma	1.04	0.93	24
**C88**	Malignant immunoproliferative diseases	1.02	0.93	392
**C90**	Multiple myeloma and malignant plasma cell neoplasms	1.03	0.93	1810
**C91**	Lymphoid leukaemia	1.11	0.31	2,662
**C92**	Myeloid leukaemia	1.43	$1.81 \cdot 10^{-12}$	2,213
**C93**	Monocytic leukaemia	1.57	0.01	815
**C94**	Other leukaemias of specified cell type	1.40	0.10	271
**C95**	Leukaemia of unspecified cell type	1.42	$3.43 \cdot 10^{-5}$	904
**C96**	Other and unspecified malignant neoplasms of lymphoid, haematopoietic and related tissue	1.19	0.93	148

eTable 2. List of diseases in the 45 most populated trajectories.

	Top 45 trajectories			Top 45 trajectories with unique patients	
ICD	Disease	Count	ICD	Disease	Count
I20	Angina pectoris	3366	I20	Angina pectoris	3366
I25	Chronic ischaemic heart disease		I25	Chronic ischaemic heart disease	
J18	Pneumonia, organism unspecified		J18	Pneumonia, organism unspecified	
E11	Non-Insulin-dependent diabetes mellitus	2514	E11	Non-Insulin-dependent diabetes mellitus	2822
E10	Insulin-dependent diabetes mellitus		E10	Insulin-dependent diabetes mellitus	
E14	Unspecified diabetes mellitus		E14	Unspecified diabetes mellitus	
H91	Other hearing loss	1280	I21	Acute myocardial infarction	2476
I20	Angina pectoris		I25	Chronic ischaemic heart disease	
I25	Chronic ischaemic heart disease		J18	Pneumonia, organism unspecified	
N40	Hyperplasia of prostate	1225	E11	Non-Insulin-dependent diabetes mellitus	2364
I10	Essential (primary) hypertension		E10	Insulin-dependent diabetes mellitus	
N30	Cystitis		J18	Pneumonia, organism unspecified	
E11	Non-Insulin-dependent diabetes mellitus	1020	I20	Angina pectoris	2294
E10	Insulin-dependent diabetes mellitus		I25	Chronic ischaemic heart disease	
H25	Senile cataract		N30	Cystitis	
I21	Acute myocardial infarction	741	H91	Other hearing loss	2171
I25	Chronic ischaemic heart disease		I20	Angina pectoris	
N30	Cystitis		I25	Chronic ischaemic heart disease	
E11	Non-Insulin-dependent diabetes mellitus	651	E11	Non-Insulin-dependent diabetes mellitus	2143
E10	Insulin-dependent diabetes mellitus		E10	Insulin-dependent diabetes mellitus	
J18	Pneumonia, organism unspecified		H25	Senile cataract	
G45	Transient cerebral ischaemic attacks and related syndromes	595	E11	Non-Insulin-dependent diabetes mellitus	2076
I63	Cerebral infarction		E10	Insulin-dependent	

				diabetes mellitus	
I69	Sequelae of cerebrovascular disease		H36	Retinal disorders in diseases classified elsewhere	
M17	Gonarthrosis [arthrosis of knee]	565	E11	Non-Insulin-dependent diabetes mellitus	2015
I10	Essential (primary) hypertension		E10	Insulin-dependent diabetes mellitus	
N30	Cystitis		N30	Cystitis	
I21	Acute myocardial infarction	460	I21	Acute myocardial infarction	1660
I25	Chronic ischaemic heart disease		I25	Chronic ischaemic heart disease	
J18	Pneumonia, organism unspecified		N30	Cystitis	
I20	Angina pectoris	454	E11	Non-Insulin-dependent diabetes mellitus	1613
I25	Chronic ischaemic heart disease		E10	Insulin-dependent diabetes mellitus	
I73	Other peripheral vascular diseases		N39	Other disorders of urinary system	
I64	Stroke, not specified as haemorrhage or infarction	437	E11	Non-Insulin-dependent diabetes mellitus	1565
J18	Pneumonia, organism unspecified		E10	Insulin-dependent diabetes mellitus	
J15	Bacterial pneumonia, not elsewhere classified		N18	Chronic renal failure	
I20	Angina pectoris	404	N40	Hyperplasia of prostate	1564
I48	Atrial fibrillation and flutter		I10	Essential (primary) hypertension	
I49	Other cardiac arrhythmias		N30	Cystitis	
J44	Other chronic obstructive pulmonary disease	375	E11	Non-Insulin-dependent diabetes mellitus	1481
J42	Unspecified chronic bronchitis		E10	Insulin-dependent diabetes mellitus	
N30	Cystitis		D64	Other anaemias	
N40	Hyperplasia of prostate	352	I20	Angina pectoris	1472
I10	Essential (primary) hypertension		I25	Chronic ischaemic heart disease	
I69	Sequelae of cerebrovascular disease		N18	Chronic renal failure	
K80	Cholelithiasis	338	E11	Non-Insulin-dependent diabetes mellitus	1406
I10	Essential (primary) hypertension		E10	Insulin-dependent diabetes mellitus	
N30	Cystitis		E16	Other disorders of pancreatic internal	

				secretion	
I20	Angina pectoris	320	I20	Angina pectoris	1369
I25	Chronic ischaemic heart disease		I25	Chronic ischaemic heart disease	
K59	Other functional intestinal disorders		I69	Sequelae of cerebrovascular disease	
E11	Non-Insulin-dependent diabetes mellitus	315	I20	Angina pectoris	1363
E10	Insulin-dependent diabetes mellitus		I25	Chronic ischaemic heart disease	
H36	Retinal disorders in diseases classified elsewhere		K59	Other functional intestinal disorders	
J44	Other chronic obstructive pulmonary disease	311	I25	Chronic ischaemic heart disease	1291
M81	Osteoporosis without pathological fracture		J18	Pneumonia, organism unspecified	
J15	Bacterial pneumonia, not elsewhere classified		J15	Bacterial pneumonia, not elsewhere classified	
I64	Stroke, not specified as haemorrhage or infarction	309	E11	Non-Insulin-dependent diabetes mellitus	1283
I61	Intracerebral haemorrhage		E10	Insulin-dependent diabetes mellitus	
I69	Sequelae of cerebrovascular disease		L97	Ulcer of lower limb, not elsewhere classified	
M17	Gonarthrosis [arthrosis of knee]	300	H91	Other hearing loss	1273
I10	Essential (primary) hypertension		I20	Angina pectoris	
N39	Other disorders of urinary system		I50	Heart failure	
F10	Mental and behavioural disorders due to use of alcohol	291	I20	Angina pectoris	1245
K70	Alcoholic liver disease		I25	Chronic ischaemic heart disease	
N30	Cystitis		I73	Other peripheral vascular diseases	
I20	Angina pectoris	278	I20	Angina pectoris	1205
I50	Heart failure		I50	Heart failure	
D64	Other anaemias		D64	Other anaemias	
I10	Essential (primary) hypertension	263	I20	Angina pectoris	1114
D64	Other anaemias		I25	Chronic ischaemic heart disease	
D63	Anaemia in chronic diseases classified elsewhere		J15	Bacterial pneumonia, not elsewhere classified	
I20	Angina pectoris	256	E11	Non-Insulin-dependent	1100

				diabetes mellitus	
K30	Dyspepsia		E10	Insulin-dependent diabetes mellitus	
I10	Essential (primary) hypertension		I69	Sequelae of cerebrovascular disease	
J44	Other chronic obstructive pulmonary disease	256	H91	Other hearing loss	1092
K59	Other functional intestinal disorders		I20	Angina pectoris	
J96	Respiratory failure, not elsewhere classified		I48	Atrial fibrillation and flutter	
N40	Hyperplasia of prostate	252	E11	Non-Insulin-dependent diabetes mellitus	1086
C61	Malignant neoplasm of prostate		E14	Unspecified diabetes mellitus	
N30	Cystitis		N39	Other disorders of urinary system	
F10	Mental and behavioural disorders due to use of alcohol	250	I21	Acute myocardial infarction	1073
I64	Stroke, not specified as haemorrhage or infarction		I25	Chronic ischaemic heart disease	
I69	Sequelae of cerebrovascular disease		I69	Sequelae of cerebrovascular disease	
E11	Non-Insulin-dependent diabetes mellitus	241	I21	Acute myocardial infarction	1067
E10	Insulin-dependent diabetes mellitus		I25	Chronic ischaemic heart disease	
N30	Cystitis		N18	Chronic renal failure	
F10	Mental and behavioural disorders due to use of alcohol	236	E11	Non-Insulin-dependent diabetes mellitus	1045
J18	Pneumonia, organism unspecified		E10	Insulin-dependent diabetes mellitus	
J15	Bacterial pneumonia, not elsewhere classified		N19	Unspecified renal failure	
E11	Non-Insulin-dependent diabetes mellitus	223	I20	Angina pectoris	1044
H25	Senile cataract		I25	Chronic ischaemic heart disease	
H26	Other cataract		E14	Unspecified diabetes mellitus	
I20	Angina pectoris	218	E11	Non-Insulin-dependent diabetes mellitus	1040
I25	Chronic ischaemic heart disease		J18	Pneumonia, organism unspecified	
N18	Chronic renal failure		J15	Bacterial pneumonia, not	

				elsewhere classified	
I10	Essential (primary) hypertension	215	I20	Angina pectoris	1036
M80	Osteoporosis with pathological fracture		I50	Heart failure	
E86	Volume depletion		N18	Chronic renal failure	
N40	Hyperplasia of prostate	204	H91	Other hearing loss	1014
I10	Essential (primary) hypertension		I20	Angina pectoris	
D64	Other anaemias		H25	Senile cataract	
M54	Dorsalgia	196	E11	Non-Insulin-dependent diabetes mellitus	1011
J44	Other chronic obstructive pulmonary disease		I70	Atherosclerosis	
M81	Osteoporosis without pathological fracture		J18	Pneumonia, organism unspecified	
H91	Other hearing loss	190	H91	Other hearing loss	995
I20	Angina pectoris		I20	Angina pectoris	
H25	Senile cataract		E78	Disorders of lipoprotein metabolism and other lipidaemias	
E11	Non-Insulin-dependent diabetes mellitus	190	N40	Hyperplasia of prostate	989
I70	Atherosclerosis		I10	Essential (primary) hypertension	
L97	Ulcer of lower limb, not elsewhere classified		I69	Sequelae of cerebrovascular disease	
I10	Essential (primary) hypertension	188	E11	Non-Insulin-dependent diabetes mellitus	986
N18	Chronic renal failure		E14	Unspecified diabetes mellitus	
K65	Peritonitis		N18	Chronic renal failure	
I20	Angina pectoris	178	M17	Gonarthrosis [arthrosis of knee]	977
I25	Chronic ischaemic heart disease		I10	Essential (primary) hypertension	
I69	Sequelae of cerebrovascular disease		N30	Cystitis	
N81	Female genital prolapse	177	I20	Angina pectoris	976
I10	Essential (primary) hypertension		I70	Atherosclerosis	
N30	Cystitis		J18	Pneumonia, organism unspecified	
C20	Malignant neoplasm of rectum	175	I25	Chronic ischaemic heart disease	964
C18	Malignant neoplasm of colon		J18	Pneumonia, organism unspecified	

C78	Secondary malignant neoplasm of respiratory and digestive organs		J96	Respiratory failure, not elsewhere classified	
E11	Non-Insulin-dependent diabetes mellitus	175	E11	Non-Insulin-dependent diabetes mellitus	961
J18	Pneumonia, organism unspecified		I70	Atherosclerosis	
J15	Bacterial pneumonia, not elsewhere classified		L97	Ulcer of lower limb, not elsewhere classified	
I64	Stroke, not specified as haemorrhage or infarction	169	I20	Angina pectoris	957
G40	Epilepsy		I25	Chronic ischaemic heart disease	
J18	Pneumonia, organism unspecified		N19	Unspecified renal failure	
I83	Varicose veins of lower extremities	160	I20	Angina pectoris	946
I50	Heart failure		I48	Atrial fibrillation and flutter	
L97	Ulcer of lower limb, not elsewhere classified		I49	Other cardiac arrhythmias	
F10	Mental and behavioural disorders due to use of alcohol	158	E10	Insulin-dependent diabetes mellitus	929
K70	Alcoholic liver disease		E14	Unspecified diabetes mellitus	
E87	Other disorders of fluid, electrolyte and acid-base balance		N18	Chronic renal failure	
